# A Molecular Communication Platform Based on Body Area Nanonetwork

**DOI:** 10.3390/nano12040722

**Published:** 2022-02-21

**Authors:** Wenxin Pan, Xiaokang Chen, Xiaodong Yang, Nan Zhao, Lingguo Meng, Fiaz Hussain Shah

**Affiliations:** School of Electronic Engineering, Xidian University, Xi’an 710071, China; wxpan@stu.xidian.edu.cn (W.P.); 18815094400@163.com (X.C.); m17865816839_2@163.com (L.M.); fsgillani@stu.xidian.edu.cn (F.H.S.)

**Keywords:** molecular communication, nanonetworks, pigment particles, body area network, platform

## Abstract

With the development of nanotechnology and biotechnology, the nanomachine can be applied to the interior of the human body. In order to achieve the goal of completing complex tasks, measures to connect multiple nanomachines that can complete more simple tasks are taken. This can expand the ability of a single nanomachine to cooperate and share information to complete more complex tasks—namely, the emergence of the Body Area Network (BAN). In response to the requirements of building a BAN, we must first need to solve the communication problem between two nanomachines. Communication networks based on molecular communication (MC), known as “natural body area networks”, are widely used in biomedical fields. With the considerable development of MC theory, it is urgent to set up an experimental platform to verify and guide theoretical modeling. In this paper, a nanomaterial-based MC platform is designed and built to simulate the cardiovascular system. The platform uses the diffusion of nanoscale pigment particles in water solution in silicone tube to achieve communication process and modulates binary sequence information to messenger molecules by on-off keying (OOK). The platform successfully transmits and receives a 17-bit binary sequence to prove its communication possibilities. To assess the platform capabilities, this paper tests the effects of different solution concentrations, pipeline flow rates, and pressure on platform communications. These factors can be used to expand the modulation schemes that the platform can implement. In future work, some nanomaterials that can be used for molecular communication can be applied to the platform to characterize their channel characteristics.

## 1. Introduction

Nanonetworks are generally considered to be the final form of current sensor networks, and Body Area Network (BAN) is considered to play an important role in the practical application of nanonetworks. In such nanonetworks, communication between devices is still an open problem [1]. In nature, ants, butterflies, bees, and many mammals use pheromones to communicate with their companions. For example, multiple glands in honeybees release pheromones made from a variety of chemicals. Different pheromones can be used in alarm, marking, attraction, aggregation, and orientation information. Inspired by the way of communication between some organisms in nature, a new multidisciplinary communication paradigm using chemical signals to transmit information has emerged—namely, molecular communication (MC) [2]. MC is very different from traditional communication methods, as follows:

- Communication carrier: In MC, information is carried by molecules, while traditional communication is carried by the electromagnetic wave, acoustic wave, or optical signal;
- Communication speed: Traditional communication based on electromagnetic waves travels much faster than MC, and has smaller memory and higher data rates;
- Communication medium: The way of wire-based communication is subject to little external influence. Air-based communication may be affected by environmental obstacles, such as tables, chairs, electrical equipment, and human bodies;
- Power consumption: In a conventional communication network, the communication process uses a battery or other external power source to achieve electrical energy. MC is generally chemically driven and has low power consumption.

Due to the size limitation of nanomachines, traditional communication technologies do not apply to communication problems between nanomachines, so that MC is more advantageous in BAN than the communication ways based on electromagnetic waves or acoustic waves. Specifically, MC has several advantages:

- Because of the size and principle of traditional acoustic sensors and radio frequency transceivers, their integration into molecular or nanoscale is not feasible. Wireless communication based on electromagnetic waves requires integrating a radio frequency transceiver in the nanomachine and achieving sufficient power, which is not easy to implement. Acoustic communication based on ultrasonic waves needs to integrate an ultrasonic sensor in nanomachine, which has a high requirement on the size of the sensor. In contrast, Molecular transceivers are essentially nanoscale and can act as nanomachines that emit and receive molecules;
- Traditional communication methods require precise physical connections between transmitters and receivers, and no direct contact between the transceiver of MC. At the same time, there is no precise navigation system in the nanomachine of the traditional communication method, which is difficult for the positioning of the receiver;
- MC can be used in situations where conventional electromagnetic wave communication is not convenient or suitable, such as in tunnels, pipes, or unpredictable underwater environments and micro/nano equipment [3].

There is much research in MC theory, which urgently needs a testbed to verify this theoretical research and guide further theoretical modeling. According to different communication media, it can be divided into MC platforms based on air [4,5,6,7] and liquid [8,9,10,11,12,13,14,15,16,17,18]. According to the channel size, the liquid-based MC platform can be divided into small pipe communication [8,9,11,13,14,15,16,17], and large pipe communication [10,12]. In 2017, Nariman Farsad et al. [9] proposed another intraductal MC platform using acid-base molecules as messenger molecules. This platform uses an aqueous solution as a communication channel and uses the pH value of the transmitted signal to represent 1-bit and 0-bit. A 0-bit is represented by an acid pulse, and a 1-bit is represented by a base pulse. The pH probe at the receiver can detect the acidity and alkalinity of the solution to determine the received information. The platform is easy to operate and has high data transfer rates. Based on the previous experiment platform, in 2020 Changmin Lee et al. [13] proposed an intraduct multi-input multi-output (MIMO) MC platform using acid-base molecules as messenger molecules to achieve a higher data rate. In 2017, Nunzio Tuccitto et al. [18] developed a remote molecular communication platform that uses concentration pulses of Carbon Quantum dots (CQDs) to encode information into binary codes. CQDs have the characteristic of self-quenching, and the relationship between fluorescence intensity and concentration is nonlinear. This paper proves that the nonlinear response is more suitable for long distance communication, and the ratio between 1 and 0 pulse is higher when the communication distance is longer. In 2020, Max Bartunik et al. [15] proposed an MC platform with ink droplets as a messenger molecule. The platform is a droplet microfluidic system that can distinguish a single droplet and the free diffusion has little effect. This platform uses a photodetector to perform droplet detection and spectral sensor to achieve droplet color detection. The platform can be extended to alternative modulation schemes, such as using droplet color, size, and length to represent different information. Harald Unterweger et al. [17] built an MC platform with superparamagnetic iron oxide nanoparticles (SPIONs). The experimental platform is excellent in the selection of messenger molecules, mainly reflected in the fact that SPIONs are chemically safe and long-term stable, medically biocompatible and clinically safe, and can be detected by magnets in a non-contact manner. In addition, the paper proposes a laminar-driven end-to-end channel pulse response model and verifies its applicability at different transmission distances and training sequences. In 2020, Bon-Hong Koo et al. [14] proposed a nano-stage MC system, which simulates communication between nanodevices in the human body. The information is encoded by the glucose molecule, and it travels through flexible tubes with nanoscale diameters, and the receiver used by the biosensor chip is implanted under the human skin to detect the concentration of glucose molecules. In 2019, Laura Grebenstein et al. [11] proposed a biological MC testbed, and the optical drive proton pump sends the *Escherichia coli* (*E. coli*) into the channel, and the pH sensor is detected as a receiver. The paper successfully demonstrates the transmission and reception of data at a time limit of 1 bit per minute. Here we introduce a large pipeline molecular communication platform. In 2019, Ladan Khaloopour et al. [12] developed a macroscopic MC platform based on fluid media. The transmitter of the platform releases hydrochloric acid, transmitted in an organic glass tube with a flow aqueous solution, and a chemical sensor and pH module as a receiving unit detect signal. Unlike microscopic MC, messenger molecules cannot be diffused alone, and additional media flow is needed to increase the throughput of the system. The paper proposes the theoretical model of the platform and verifies its correctness, studying the error rate and communication rate of the system.

The main contributions of this paper are as follows:

1. In this paper, an MC platform based on a single power source is designed and built. The transmitter combines a peristaltic pump with electromagnetic valves to inject water of different colors into a silicone tube that simulates the vascular environment in the body. The color sensor acts as a receiver to detect the signal;
2. To reduce interference in a communication channel, different sensors are used to characterize some parameters of the molecular communication system, enabling non-contact measurements of pressure and flow rate;
3. In this paper, the communication feasibility of the molecular communication platform is proved, and the influence of concentration, flow rate, and pressure on the communication channel and signal reception is measured.

The remainder of this article is organized as follows. In Section 2, the MC experiment platform and the communication protocol used are presented. Section 3 assesses the capacity of the MC platform when the concentration, flow rate, and pressure change. Finally, Section 4 concludes this article and provides a direction for the future.

## 2. Platform

Building a relatively complete molecular communication laboratory requires five parts: modulation, demodulation, transmission, propagation, and receiving. Modulation refers to how to carry binary sequence information on messenger molecules. Transmission refers to how to carry information, provide power and send molecules to the propagation channel. The propagation refers to transmitting the messenger molecules from the transmitting end through the medium to the receiving end. Receiving refers to detection of the messenger. The demodulation is the inverse process of the modulation, and extracts the information expressed in the received solution.

In traditional communication, information is encoded by binary, while in molecular communication, information is encoded on the molecule itself. One or more physical characteristics of messenger molecules are utilized to encode information in a variety of combinations, including nature, type, time, space, and mixed modulation [19]. Property-based modulation uses different physical and chemical properties of messenger molecules to represent information, such as concentration, optical properties, magnetism, reactivity, etc. Molecular type-based modulation uses different types of messenger molecules to represent different messages. Time-based modulation means that different release times of messenger molecules are used to represent different messages. Spatial modulation refers to information represented by the spatial position of the transmission and is mainly used in MIMO systems. Mixed modulation refers to the combination of the above modulation modes. Common modulation methods are concentration modulation because relative to other modulation methods, its technical complexity is the lowest, the equipment requires the lowest, easy to achieve synchronization. The simplest concentration modulation method is OOK; when the concentration of the messenger molecules is 0, it represents 0-bit, and when the concentration of the messenger molecules is not zero, it represents 1-bit.

The propagation medium is mainly divided into air and liquid. In the air, molecules can spread through free diffusion, and in flowing aqueous solution, they disperse passively continuously, or discretely in channels. Continuous diffusion occurs if messenger molecules can be soluble in the background aqueous solution at the macro level, and discrete diffusion occurs if messenger molecules are insoluble in background flow droplets or other substances. In the process of air-based free diffusion, it is necessary to pay attention to the possibility that some of the messenger molecules may spread out of the expected channel range, resulting in the loss of molecules reaching the receiver, while in addition, it may be expected that out-of-channel molecules will drift into the channel and be received by the receiving end, which will affect the communication results.

**Figure 1:**
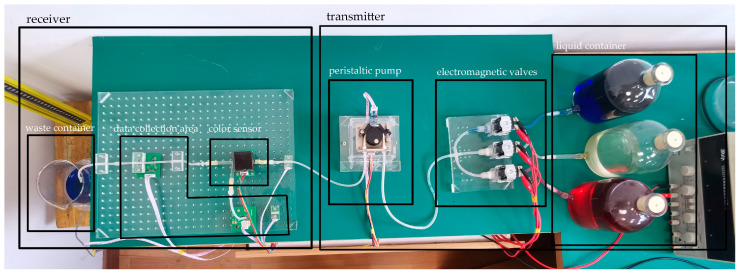
MC platform.

Before setting up the experimental platform, the communication scheme of the whole system needs to be determined. Therefore, this section introduces the preconceived communication protocol of the system, and then introduces the components of the molecular communication system, as shown in Figure 1. It consists of three parts: transmitter, channel, and receiver.

### 2.1. Theoretical Model

According to the messenger molecules propagating from the transmitting end to the receiving end, molecular communication can be divided into two main types: the first type is a passive molecule communication, and the second type is active molecule communication. Among them, in passive molecular communication, the molecule is freely diffused from the transmitting end to the receiving end and does not require an intermediate system to transmit the molecule. The entire molecular communication model includes transmitting, diffusion, and receiving. This article focuses on channel characterization, so this part focuses on the molecular communication diffusion model.

The free diffusion model of the molecule is a core basic model of molecular communication. As long as the transmitter releases the messenger molecule into the fluid channel, the messenger molecule will diffuse in the fluid medium. Messenger molecules suspended in the liquid are forever in an irregular motion, called Brownian motion, as they are bombarded by neighboring molecules. In order to describe the location distribution of diffusion molecules, Fick established in 1855 an equation describing the diffusion of concentration from high to low, known as Fick’s First law. Considering only the *X*-axis, the mathematical expression of Fick’s first law in one-dimensional state is as follows:

(1) $$J_{X}=-D\frac{\partial C}{\partial x},$$

where *J_x_* and *C* are the diffusion flux and concentrations of the molecules in point *x*, respectively. *D* is a diffusion coefficient, which is a constant, the diffusion coefficient is usually related to factors such as temperature, fluid viscosity, and molecules. According to Fick’s First law, messenger molecules spread from a high concentration to a low concentration. In order to describe the form of concentration changing with time at a certain point, Fick’s second law is further deduced on the basis of Fick’s first law. The one-dimensional diffusion equation of Fick’s second law is mathematically expressed as follows:

(2) $$\frac{\partial C}{\partial t}=D\frac{\partial^{2}C}{\partial x^{2}},$$

it is easy to prove that the solution of the one-dimensional diffusion equation is

(3) $$C=\frac{K}{\sqrt{t}}e^{-\frac{x^{2}}{4Dt}}$$

Suppose that in the free diffusion model of molecular communication, the transmitter releases *M* messenger molecules at the origin of coordinates at *t* = 0. With the help of the above equation, it can be obtained:

(4) $$M=2k\sqrt{\pi D},$$

(5) $$C=\frac{M}{\sqrt{4\pi Dt}}e^{-\frac{x^{2}}{4Dt}}$$

In order to describe the distribution of messenger molecules in space, the diffusion equation extended to three dimensions is:

(6) $$C=\frac{K}{t^{\frac{3}{2}}}e^{\frac{-\left(x^{2}+y^{2}+z^{2}\right)}{4Dt}}$$

According to the method of solving one-dimensional diffusion equation, the solution of three-dimensional diffusion equation is [20]:

(7) $$C=\frac{M}{{\left(4Dt\right)}^{\frac{3}{2}}}e^{\frac{-\left(x^{2}+y^{2}+z^{2}\right)}{4Dt}}$$

As can be seen from the diffusion model of molecular communication, in the process of transmission from the transmitter end to the receiver end, the messenger molecules do free diffusion, and the messenger molecules emitted by the transmitter end will freely diffuse to the receiver end with low concentration. According to the above equations, it can be concluded qualitatively that the farther the diffusion distance is, the molecular concentration will decrease. This laid the foundation for building a molecular communication platform.

### 2.2. Communication Protocol

First of all, this system adopts a binary OOK modulation scheme based on the concentration; that is, colored stands for bit 1, colorless stands for bit 0. According to [21], the effects of full pulse transmission and pulse width reduction on inter-symbol interference (ISI) are different. Therefore, in this system, a unit time slot is defined as 10 s, in a unit time slot; if sending 10 s of colorless water solution, it means sending 0; if sending part of the colored solution, it means sending 1. The pigment molecule is chosen as the carrier of information in the system, because the pigment is easy to obtain and the cost is low, and the observed effect is obvious. To simulate the human cardiovascular system and the human heart pumping blood, the delivery of messenger molecules to choose peristaltic pump to provide power. After the discussion in the previous section, the system chooses to use the flow-assisted diffusion mode to transmit the signal, and the peristaltic pump provides the power to input the background flow continuously. The choice of the receiver depends on the messenger molecules and the modulation mode. Because the messenger molecule is the pigment molecule and adopts OOK modulation mode, the color sensor is used as the receiver to detect the color change of the solution at the receiver. Demodulation is the reverse process of modulation and the process of recovering a message from a modulated signal carrying the message. Specifically, the demodulation method of the system is to judge that the solution at the receiving position has color to indicate receiving bit 1, colorless to indicate receiving bit 0. A complete communication process is: the encoded binary sequence is modulated on the pigment molecule, and the pigment molecule is sent to the catheter channel by the transmitter and transmitted to the color sensor at the receiver, then, the binary sequence information is demodulated according to the data displayed by the color sensor.

### 2.3. Transmitter

The transmitter includes a peristaltic pump, three liquid containers, and corresponding three electromagnetic valves. Red solutions, blue solutions, and colorless aqueous solutions are filled in three liquid containers. The red solution and blue solution were made of 0.2 g bright red pigment and 0.2 g bright blue pigment in 1 L water. It should be noted that the pigments used herein are water-soluble and will not remain in the silicone tube, affecting the determination of subsequent parameters. The red solution was used as the control group. Under the same conditions, we replaced the blue solution with the red solution for the control experiment to ensure the robustness and repeatability of the system. The experiment designed in this paper mainly uses blue solution and colorless solution. In the next stage, blue, red and colorless solutions can be considered to achieve multistage modulation.

To simulate the human heart and gastrointestinal system, the molecular communication system uses a constant speed peristaltic pump to provide power. The peristaltic pump voltage is 24 V, and the maximum flow rate is 154 mL/min. The speed of the peristaltic pump is controlled by a single chip microcomputer to synchronize with the data collection area. The core of the peristaltic pump is a stepper motor, severe shaking will occur in the process of use, so another customized acrylic box to fix the peristaltic pump, reduce the effect of shaking on the experiment. To avoid the peristaltic pump’s setting speed and the actual speed not matching, use magnets and a Hall sensor to measure the true speed of the peristaltic pump. The magnet is attached to the peristaltic pump, which turns the magnet; the Hall sensor is attached to the clasp of the peristaltic pump. The speed of peristalsis is determined by measuring the time it takes for the magnet to rotate once using a Hall sensor, as shown in the diagram.

The peristaltic pump in this experiment is working continuously to maintain the power of the whole system, so it needs another piece of equipment to control the sending of colored solution or colorless solution, power is 4 W. The three electromagnetic valves are connected to three liquid containers containing blue solution, colorless water solution, and red solution, as electromagnetic valve A, electromagnetic valve B, and electromagnetic valve C. In addition, two driving modules are designed to drive the electromagnetic valve. The output voltage of the driving module is between 5–35 V and the maximum driving current is 2 A. It is important to note that the three electromagnetic valves cannot be open at the same time. Because colorless water solution is the role of the background flow, electromagnetic valve B is always open; when we need to inject the blue solution, we close electromagnetic valve B, then open electromagnetic valve A.

### 2.4. Propagation Channel

Silicone tubes with an inner diameter of 2 mm and an outer diameter of 4 mm were used to simulate the vascular system in the human body. The platform uses these silicone tubes because they are inexpensive and can be easily connected, bent, and placed to form complex networks. More importantly, the silicone tube is similar to the biological tissue. The diffusion of the aqueous solution containing pigment molecules alone will lead to a slow transmission rate, so the continuous flow of colorless aqueous solution is added in the channel as the background flow, which is also consistent with the continuous flow of blood in the human body. In [22], various parameters of molecular communication in human blood and water are very similar, and human blood can be replaced by water. Therefore, this paper adopts water as background flow to simulate human blood.

### 2.5. Receiver

In order to more accurately observe the color change of the solution at the receiver, the silica gel tube is connected to a glass tube of higher transparency and the same size as the silicone tube. A color sensor is placed underneath the glass tube to identify the color of the solution inside the glass tube. The voltage of the color sensor is 2.7–5.5 V, and it contains 64 photodiodes, including 16 colorless filters, 16 red filters, 16 blue filters, and 16 green filters. These four filter photodiodes correspond to four data channels. Among them, the red filter channel, green filter channel, and blue filter channel can obtain the values of R, G, and B of the solution color, respectively. In order to make the measurement data more accurate, the acrylic roof design, glass tube, and color sensor position are fixed. The color sensor comes with 4 luminous LED lights to provide the light source. In order to avoid interference from external light, a small black box is made to cover the color sensor. It should be noted that white balance should be carried out on the color sensor before each experiment; that is, to tell the system what is white, so as to identify the color more accurately. The aqueous solution flows from the liquid container to the receiving end and finally into the waste liquid collection bottle.

**Figure 2:**
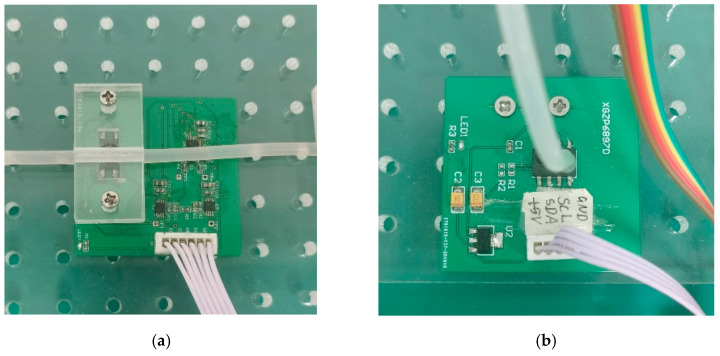
These are two sensors that measure pressure in the pipe. (**a**) The contact force sensor for measuring the internal pressure of the glass tube; (**b**) The pressure sensor for measuring the pressure of silicone tube.

Since the molecular communication system in this paper simulates the vascular environment of the human body, the parameters of the molecular communication system need to meet the conditions of non-contact and be easy to obtain. In order to measure the flow velocity in the MC system and meet the condition of non-contact, common flow meters cannot be used. In this paper, the weighing sensor is selected, and the precision of the calibrated weighing sensor is 0.1 g. The flow rate of the system can be measured by periodically sampling the weight of the waste container with a weighing sensor. In order to measure the pressure in the flexible material silicone tube, we choose the contact force sensor, whose power supply voltage is 5 V, and determine the pressure in the silicone tube by measuring the extrusion of the silicone tube to the contact force sensor. The contact force sensor is shown in Figure 2a. It should be noted that, due to the existence of the peristaltic pump, the silicone tube will shake vigorously during the experiment, which affects the measurement of the contact force sensor. Therefore, the self-made acrylic plate is used to fix the silicone tube. It is not appropriate to use a contact force sensor to measure the pressure in a rigid material glass tube, so a T-shaped glass tube and a pressure sensor are used, as shown in Figure 2b. Pathway 1 is a liquid pathway, and pathway 2 is a gas pathway. Tilt the T-shaped glass tube slightly to prevent flow in pathway 2. Pathway 2 is connected to a pressure sensor to determine the pressure of the liquid in pathway 1 by measuring the pressure of the gas in pathway 2.

## 3. Experimental Results and Discussion

This section proves the communication feasibility of the platform and gives some experimental data and analysis measured on the experiment. Among them, the communication feasibility of the first part is the test construction, the second part tests the effects of different blue solution concentrations on communication, and the third part is the effect of different flow rates and pressure to communicate.

### 3.1. Communication Feasibility

**Figure 3:**
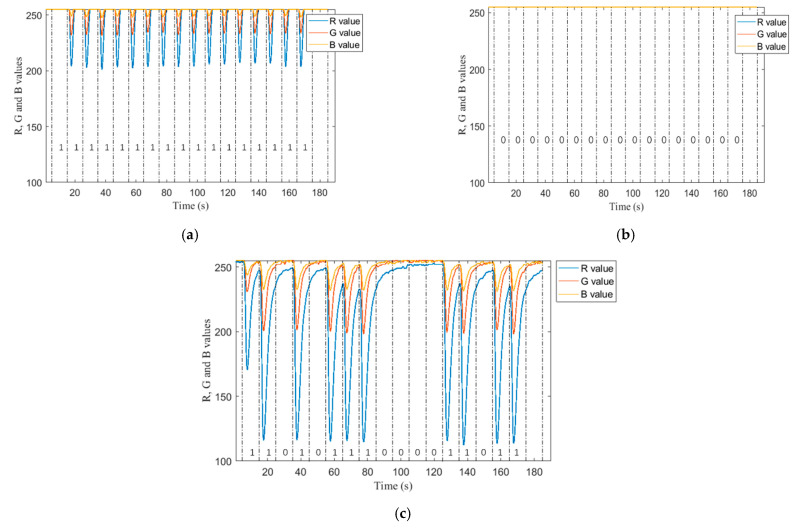
R, G and B values when sending different sequences. The background flow rate is set to 66 mL/min, the k value is 0.05. (**a**) R, G and B values when sending 17-bit full 1 sequence; (**b**) R, G and B values when sending 17-bit full 0 sequence; (**c**) R, G and B values when sending 17-bit {1,1,0,1,0,1,1,0,0,0,0,0,1,1,0,1,1,1,1,1,1} sequence.

As mentioned above, the ISI can be reduced by appropriately reducing the pulse width. Therefore, we first define k as the proportion of sending blue solution time in a unit time slot. For example, when k is 0.1, it represents sending 1 s blue solution and 9 s colorless solution per unit time slot. To verify the feasibility of communication, the R, G, and B values measured by the color sensor are tested respectively when sending 17-bit full 1 sequence, 17-bit full 0 sequence and 17-bit {1,1,0,1,0,1,1,0,0,0,0,0,1,1,0,1,1,1,1,1,1} sequence, as shown in Figure 3, the R, G and B values measured by the color sensor represent a unit time slot between each of the two dotted lines. Here’s how it works:

1. When the R, G, and B values are 255, it indicates that the solution flowing through the color sensor is colorless; that is, 0-bit is received; When the flowing color sensor is a blue solution, it shows different RGB values.
2. Since the blue solution is used, the value of R change is most obvious. To simplify the experiment, the G and B values can be omitted, and only the change of the value of R is displayed.
3. Select a certain threshold value of R. If the value is higher than this threshold, 0 is received properly. If the value is lower than this threshold, 1 is received properly. Based on this rule, it can be found that the first bit 1 sent is detected as bit 0 when sending the whole 1 sequence, and when sending the sequence {1,1,0,1,0,1,1,1,0,0,1,1,1,1}, the R of the first bit sent is not as variable as the other bits. In addition, after many experiments sending other different sequences, it was also found that the first bit did not change significantly. So, the first bit is not treated as the communication bit to ensure the accuracy of the communication.

**Figure 4:**
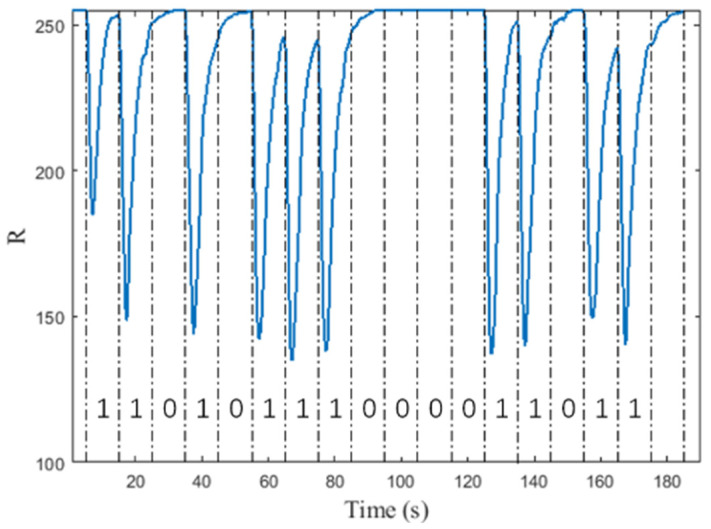
Example of communication.

After multiple trials, a fixed threshold 210 is provided for the value of R, and if R value is less than the threshold for more than 30% of the time in a single time slot, the symbol received in the current time slot is considered to be 1-bit; otherwise, it is 0-bit. After discussion, the communication feasibility of the testbed is proved. Here is an example of communication, as shown in Figure 4. The input sequence is 17 bits {1,1,0,1,0,1,1,1,0, 0,0,0,1,1,0,1,1}; discard the first bit and do not use it as a communication bit. The background flow rate is set to 66 mL/min; the k value is 0.05.

### 3.2. Different Concentrations

**Figure 5:**
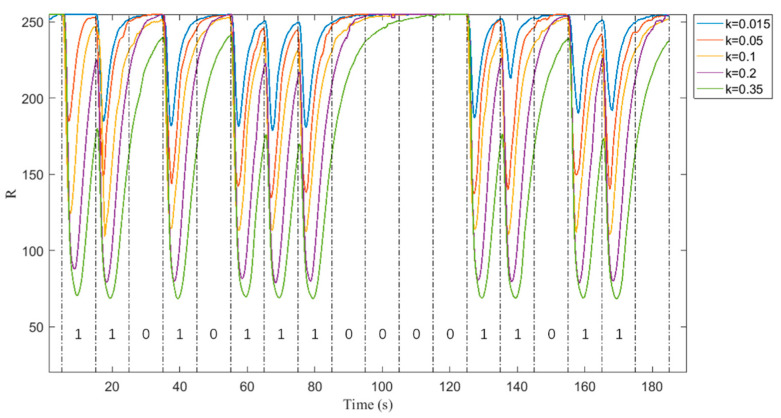
R value at different concentrations.

A different proportion of blue solution in the unit time slot means different concentrations of the blue solution; different concentrations will affect the quality of communication. A higher concentration will lead to more influence of ISI. Lower concentrations can make it difficult for color sensors to detect color changes. Because blue pigment molecules in higher concentrations can remain in the tube too late to diffuse and affect the next time slot, ISI is greater. This paper aims to minimize the use of pigment particles and distinguish different communication bits. In order to balance the two, it is very important to select the appropriate concentration. All other things being equal, the R values measured by the color sensor at different k values (k = 0.015, 0.05, 0.1, 0.2, 0.35) are compared, as shown in Figure 5. As can be seen from the diagram, the larger the k value, the wider the pulse width, and the greater the change of R value. By observing the blue line in Figure 5, it can be concluded that if the k value is too small, the color sensor will not recognize the first 1 bit sent, which greatly increases the bit error rate. This is because the blue concentration is too small, and the color is too light. The switch between the open and closed state of the solenoid valve is not instantaneous, resulting in the uncertainty of the concentration of the solution and the channel state during this time. Therefore, in order to ensure communication quality, it is necessary to select the appropriate concentration of the blue solution. In addition, this leads to a new way of encoding; that is, using different concentrations to represent different signals.

### 3.3. Different Pressure and Flow Rates

**Figure 6:**
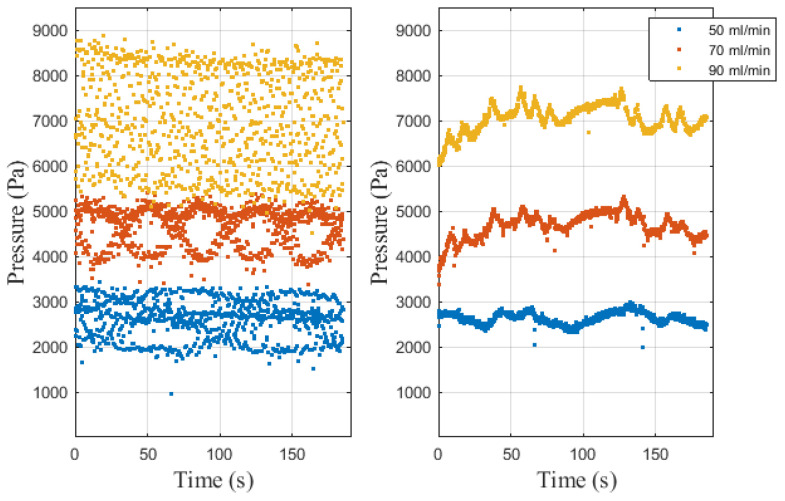
Measured pressure in the pipe. The left side is the measurement of the air pressure sensor, and the right side is the measurement of the touch sensor.

There is a significant difference in blood flow and blood pressure in different genders, ages, and physical index groups. In addition, human flow and blood pressure will change in the case of vigorous exercise or emotional excitement. The flow rate and pressure in this system are correlated with the blood flow and blood pressure of the human body, so it is necessary to study the influence of flow rate and pressure on the molecular communication system. Changes in human blood flow and blood pressure are generated by the heart pump, so in the molecular communication system in this paper, pressure and flow rate in the tube can be changed by changing the peristaltic pump speed. Firstly, the method of measuring pressure in this system is introduced. To increase the robustness of the system, the pressure is measured in two ways. The pressure inside the glass tube and the pressure inside the silicone tube are measured by the air pressure sensor and the touch force sensor, respectively, as shown in Figure 6. It can be seen that the pressure varies greatly at different flow rates, and the higher the flow rate is, the higher the pressure in the tube. Comparing the left and right pictures in Figure 6, it can be seen that the pressure jitter measured by the touch force sensor is relatively small because the silicone tube measured by the contact force sensor is fixed by the acrylic block to reduce the jitter.

**Figure 7:**
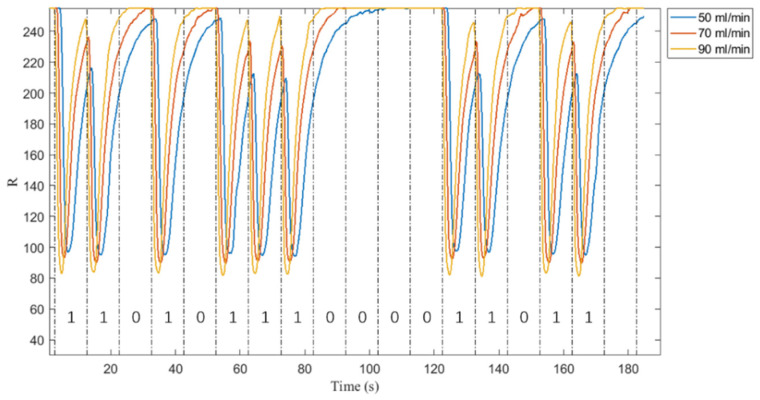
R values at different flow rates.

To simulate the human internal environment, different flow rate groups were set in the system: 50 mL /min, 70 mL/min, and 90 mL/min [23,24]. Figure 7 shows the R value at different flow rates. It can be seen from Figure 7 that the rate of change of R value increases with the increase of flow rate. This is because the higher the flow rate, the more solution in the unit time slot, and the darker the color at 1 bit. In addition, the larger the velocity is, the smaller the pulse width and the smaller the ISI. In order to reduce ISI, it is very important to select the appropriate velocity.

## 4. Conclusions

This paper aims to build a molecular communication platform that can be used in nanomaterials, focusing on the construction of a nanomaterial-based platform and the measurement of actual data. In this experimental platform, nanoscale pigment particles, which are easy to obtain and low-cost, are selected as the carrier of information, and binary sequences are modulated on messenger molecules by using the OOK method. The peristaltic pump, which simulates the human heart, is used to provide the power to send the solution with messenger molecules to the silicone tube channel. A color sensor acting as a receiver detects the color of the transmitted solution to demodulate it out of binary sequence information. In this paper, the communication feasibility of the platform is verified, and some channel parameters such as pressure and flow rate can be measured without contact. For new nanomaterials, after computer simulation to obtain the optimal solution, these nanomaterials can be applied to the platform, the combination of theory and practice to improve the research of nanomaterials.

## Data Availability

Not applicable.

## References

[1] Yang K., Bi D., Deng Y., Zhang R., Rahman M.M.U., Ali N.A., Imran M.A., Jornet J.M., Abbasi Q.H., Alomainy A. (2020). A Comprehensive Survey on Hybrid Communication in Context of Molecular Communication and Terahertz Communication for Body-Centric Nanonetworks. IEEE Trans. Mol. Biol. Multi-Scale Commun..

[2] Farsad N., Yilmaz H.B., Eckford A., Chae C.-B., Guo W. (2016). A Comprehensive Survey of Recent Advancements in Molecular Communication. IEEE Commun. Surv. Tutor..

[3] Hiyama S., Moritani Y., Suda T., Egashira R., Enomoto A., Moore M., Nakano T. Molecular Communication. Proceedings of the 2005 NSTI Nanotechnology Conference and Trade Show—NSTI Nanotech 2005 Technical Proceedings.

[4] Farsad N., Guo W., Eckford A. (2013). Tabletop Molecular Communication: Text Messages through Chemical Signals. PLoS ONE.

[5] Giannoukos S., Marshall A., Taylor S., Smith J. (2017). Molecular Communication over Gas Stream Channels Using Portable Mass Spectrometry. J. Am. Soc. Mass Spectrom..

[6] Shakya P., Kennedy E., Rose C., Rosenstein J.K. (2018). Correlated Transmission and Detection of Concentration-Modulated Chemical Vapor Plumes. IEEE Sens. J..

[7] Damrath M., Bhattacharjee S., Hoeher P.A. (2021). Investigation of Multiple Fluorescent Dyes in Macroscopic Air-Based Molecular Communication. IEEE Trans. Mol. Biol. Multi-Scale Commun..

[8] Krishnaswamy B., Austin C.M., Bardill J.P., Russakow D., Holst G.L., Hammer B.K., Forest C.R., Sivakumar R. (2013). Time-Elapse Communication: Bacterial Communication on a Microfluidic Chip. IEEE Trans. Commun..

[9] Farsad N., Pan D., Goldsmith A. A Novel Experimental Platform for In-Vessel Multi-Chemical Molecular Communications. Proceedings of the GLOBECOM 2017—2017 IEEE Global Communications Conference.

[10] Atthanayake I., Esfahani S., Denissenko P., Guymer I., Thomas P.J., Guo W. (2018). Experimental Molecular Communications in Obstacle Rich Fluids. Proceedings of the 5th ACM International Conference on Nanoscale Computing and Communication.

[11] Grebenstein L., Kirchner J., Wicke W., Ahmadzadeh A., Jamali V., Fischer G., Weigel R., Burkovski A., Schober R. (2019). A Molecular Communication Testbed Based on Proton Pumping Bacteria: Methods and Data. IEEE Trans. Mol. Biol. Multi-Scale Commun..

[12] Khaloopour L., Rouzegar S.V., Azizi A., Hosseinian A., Farahnak-Ghazani M., Bagheri N., Mirmohseni M., Arjmandi H., Mosayebi R., Nasiri-Kenari M. (2019). An Experimental Platform for Macro-Scale Fluidic Medium Molecular Communication. IEEE Trans. Mol. Biol. Multi-Scale Commun..

[13] Lee C., Koo B.-H., Chae C.-B., Chen Y., Nakano T., Lin L., Mahfuz M.U., Guo W. (2020). Molecular MIMO Communications Platform with BTSK for In-Vessel Network Systems. Bio-inspired Information and Communication Technologies; Lecture Notes of the Institute for Computer Sciences, Social Informatics and Telecommunications Engineering.

[14] Koo B., Kim H.J., Kwon J., Chae C. Deep Learning-Based Human Implantable Nano Molecular Communications. Proceedings of the ICC 2020—2020 IEEE International Conference on Communications (ICC).

[15] Bartunik M., Fleischer M., Haselmayr W., Kirchner J. (2020). Colour-Specific Microfluidic Droplet Detection for Molecular Communication. Proceedings of the 7th ACM International Conference on Nanoscale Computing and Communication.

[16] Wicke W., Unterweger H., Kirchner J., Brand L., Ahmadzadeh A., Ahmed D., Jamali V., Alexiou C., Fischer G., Schober R. (2021). Experimental System for Molecular Communication in Pipe Flow with Magnetic Nanoparticles. arXiv.

[17] Bicen A.O., Austin C.M., Akyildiz I.F., Forest C.R. (2015). Efficient Sampling of Bacterial Signal Transduction for Detection of Pulse-Amplitude Modulated Molecular Signals. IEEE Trans. Biomed. Circ. Syst..

[18] Tuccitto N., Li-Destri G., Messina G.M.L., Marletta G. (2017). Fluorescent Quantum Dots Make Feasible Long-Range Transmission of Molecular Bits. J. Phys. Chem. Lett..

[19] Kuran M.Ş., Yilmaz H.B., Demirkol I., Farsad N., Goldsmith A. (2021). A Survey on Modulation Techniques in Molecular Communication via Diffusion. IEEE Commun. Surv. Tutor..

[20] Crank J. (1979). The Mathematics of Diffusion.

[21] Mahfuz M.U., Makrakis D., Mouftah H.T. Characterization of Intersymbol Interference in Concentration-Encoded Unicast Molecular Communication. Proceedings of the 2011 24th Canadian Conference on Electrical and Computer Engineering (CCECE).

[22] Mahfuz M.U., Makrakis D., Mouftah H.T. On the Characteristics of Concentration-Encoded Multi-Level Amplitude Modulated Unicast Molecular Communication. Proceedings of the 2011 24th Canadian Conference on Electrical and Computer Engineering (CCECE).

[23] Zhu D.N., Wang T.K. (2018). Physiology.

[24] Zhou H. (2014). Research of the Blood Flow Velocity Measurement Technology Based on Optical Fiber Sensing Technology. Ph.D. Thesis.

